# Metabolic Multimorbidity and Acute Obstructive Presentation in Colon Cancer: A 677-Patient Hospital-Based Cohort

**DOI:** 10.3390/jcm15010038

**Published:** 2025-12-20

**Authors:** Lucian-Flavius Herlo, Octavian Marius Creţu, Alexandra Herlo, Danut Dejeu, Aneta-Rada Dobrin, Adelina Raluca Marinescu, Talida Georgiana Cut, Claudia Raluca Balasa Virzob, Radu Gheorghe Dan, Raluca Dumache

**Affiliations:** 1 Doctoral School, “Victor Babes” University of Medicine and Pharmacy Timisoara, 300041 Timisoara, Romania; flavius.herlo@umft.ro (L.-F.H.); aneta.goia@umft.ro (A.-R.D.); adelina.marinescu@umft.ro (A.R.M.); talida.cut@umft.ro (T.G.C.); 2 Department of Surgical Semiology, “Victor Babes” University of Medicine and Pharmacy Timisoara, 300041 Timisoara, Romania; octavian.cretu@umft.ro (O.M.C.); radu.dan@umft.ro (R.G.D.); 3 Department XIII, Discipline of Infectious Diseases, “Victor Babes” University of Medicine and Pharmacy Timisoara, 300041 Timisoara, Romania; 4 Surgical Oncology Department, Emergency County Hospital Oradea, 410169 Oradea, Romania; 5 Department of Clinic Nursing, “Victor Babes” University of Medicine and Pharmacy Timisoara, 300041 Timisoara, Romania; virzob.claudia@umft.ro; 6 Department of Forensic Medicine, Bioethics, Medical ethics and Medical Law, “Victor Babes” University of Medicine and Pharmacy Timisoara, 300041 Timisoara, Romania; raluca.dumache@umft.ro; 7 Center for Ethics in Human Genetic Identifications, “Victor Babes” University of Medicine and Pharmacy Timisoara, 300041 Timisoara, Romania

**Keywords:** colorectal neoplasms, metabolic syndrome, comorbidity, intestinal obstruction, length of stay

## Abstract

**Background/Objectives**: Metabolic comorbidities and systemic inflammation are implicated in colon carcinogenesis, yet their relationship with acute obstructive presentation and early in-hospital course remains unclear. This study evaluated whether age, metabolic multimorbidity, and inflammatory–metabolic biomarkers are associated with obstruction severity and length of stay in a surgical colon cancer cohort. **Methods**: We analyzed 677 consecutive adults undergoing surgery for histologically confirmed colon cancer. Acute presentation was categorized as no obstruction, subocclusive syndrome, or frank obstruction. Predictors included age, comorbidity count (multimorbidity defined as ≥2), diabetes, hypertension, and preoperative biomarkers (C-reactive protein (CRP), lipids, glucose; neutrophil-to-lymphocyte ratio (NLR)/platelet-to-lymphocyte ratio (PLR)/C-reactive protein-to-albumin ratio (CAR)where available). Multivariable logistic and ordinal regression assessed obstructive presentation; linear regression assessed length of stay. **Results**: Subocclusion or obstruction occurred in 34.8% of patients. In multivariable logistic regression, age was independently associated with obstructive presentation (odds ratio (OR) 1.016 per year; 95% confidence interval (CI) 1.001–1.032), while comorbidity count and CRP were not. In an ordinal model, age increased the odds of more severe presentation (OR 1.018 per year), whereas diabetes was inversely associated (OR 0.573). Length of stay was independently associated only with presentation severity (β = −0.959 days per category). Correlations between inflammatory indices and length of stay were negligible. **Conclusions**: In this hospital-based surgical cohort, age showed a modest association with obstructive presentation, while metabolic multimorbidity and routine inflammatory markers provided limited discrimination for obstruction or early in-hospital resource use.

## 1. Introduction

Colorectal cancer (CRC) is among the most commonly diagnosed malignancies worldwide and remains a leading cause of cancer-related death, with almost two million new cases and one million deaths estimated in 2020 [1,2]. Global burden analyses show that both incidence and mortality have risen steadily over recent decades, particularly in regions undergoing rapid socioeconomic transition, and predict further increases as populations age and westernized lifestyle patterns become more prevalent [2,3]. These trends position colorectal cancer at the intersection of demographic aging and modifiable metabolic and behavioral risk factors [4,5,6].

A substantial proportion of colorectal cancers is attributable to lifestyle-related exposures. Syntheses from the World Cancer Research Fund/American Institute for Cancer Research Continuous Update Project indicate that maintaining a healthy body weight, engaging in regular physical activity, consuming a predominantly plant-based diet and limiting alcohol and processed meat intake substantially lower colorectal cancer risk [6,7,8,9,10]. Conversely, obesity, type 2 diabetes, hypertension, dyslipidemia and non-alcoholic fatty liver disease cluster with these behaviors and have been consistently linked to increased colorectal cancer incidence and, in some cohorts, to earlier-onset disease [11,12,13,14,15,16,17,18]. At the biological level, colon carcinogenesis typically follows the classical adenoma–carcinoma sequence, in which stepwise accumulation of genetic and epigenetic alterations in adenomatous polyposis coli (APC), Kirsten rat sarcoma viral oncogene homolog (KRAS), tumor protein p53 (TP53) and related pathways drives progression from benign adenoma to invasive carcinoma [7]. In parallel, a serrated neoplasia pathway—characterized by v-Raf murine sarcoma viral oncogene homolog B1 (BRAF) mutations, CpG island methylator phenotype (CIMP) and frequent MLH1 promoter hypermethylation—gives rise to a substantial subset of right-sided and microsatellite-unstable cancers [8]. These molecular trajectories are strongly modulated by pro-carcinogenic exposures in the colonic lumen and mucosa, including carcinogens formed during high-temperature cooking of meats, heme iron, alcohol-derived acetaldehyde, and tobacco-related nitrosamines, as well as by chronic inflammation and oxidative stress [4,6,8]. Increasing evidence also implicates microbiome dysbiosis in this process: high intra-tumoral levels of *Fusobacterium nucleatum*, for example, are associated with more advanced T stage, distant metastasis, poor differentiation, and worse overall and disease-free survival, suggesting that specific microbial signatures may both reflect and promote an aggressive tumor phenotype [9].

Metabolic comorbidities such as obesity, type 2 diabetes, hypertension, dyslipidemia, and non-alcoholic fatty liver disease cluster tightly with these lifestyle exposures and can be regarded as downstream phenotypes of unhealthy diet and inactivity. A meta-analysis of observational studies reported that individuals with metabolic syndrome have approximately 30–40% higher risk of colorectal cancer compared with metabolically healthy counterparts [10]. Likewise, diabetes mellitus confers about a 30% increase in colorectal cancer risk, independent of body mass index, potentially through chronic hyperinsulinemia, activation of the insulin-like growth factor axis, and low-grade systemic inflammation [11]. An umbrella review synthesizing nearly 50 meta-analyses further confirmed robust positive associations between colorectal cancer risk and obesity, diabetes, hypertension, metabolic syndrome, and non-alcoholic fatty liver disease, with summary relative risks in the range of 1.2–1.6 for these exposures [12]. Beyond incidence, these metabolic conditions are linked to more advanced tumor stage at diagnosis, altered tumor biology, and potentially worse tolerance of surgery and systemic therapies, underscoring metabolic health as both a marker and mechanistic contributor to colon carcinogenesis [10,11,12].

These trends are particularly concerning in younger adults. Several high-quality cohorts and systematic reviews have documented a rising incidence of early-onset colorectal cancer (<50 years), especially in high-income settings where obesity, sedentary behavior, and ultra-processed, Western-style diets are increasingly prevalent [2,3]. In the Nurses’ Health Study II, women with obesity (body mass index ≥ 30 kg/m^2^) had nearly double the risk of early-onset colorectal cancer compared with lean women, and large weight gain since young adulthood was associated with further risk elevation [13]. A systematic review of 26 studies on early-onset colorectal cancer identified processed meat, sugar-sweetened beverages, Western dietary patterns, excess body weight, low physical activity and smoking as recurrent, modifiable risk factors, with emerging roles for epigenetic alterations and microbiome-mediated mechanisms as downstream mediators [14].

Clinically, a substantial proportion of patients still present late with complications of luminal narrowing, including sub-occlusive syndrome and frank intestinal obstruction, particularly in settings with suboptimal screening coverage and high burden of metabolic disease. Emergency or urgent surgery for obstructing colon cancer is consistently associated with more advanced tumor stage, greater physiological derangement, and adverse short-term outcomes. In a recent single-center analysis of colon cancer surgery, emergency cases had significantly poorer in-hospital survival (approximately 75% vs. substantially higher survival after elective surgery), higher rates of ostomy formation, and longer hospital stays, alongside higher preoperative systemic inflammatory markers such as neutrophil-to-lymphocyte and platelet-to-lymphocyte ratios [15]. Obstructive presentation in colon cancer is driven not only by host factors but also by tumor-specific characteristics. Left-sided and sigmoid lesions, circumferential or annular growth patterns, and bulky, high T-stage tumors are more likely to produce luminal narrowing and acute obstruction, whereas right-sided or exophytic lesions may reach considerable size before causing mechanical compromise. In parallel, diagnostic routes and screening coverage strongly influence whether cancers are detected at an asymptomatic stage or only when obstructive symptoms develop [12,13,14,15].

In this context, we analyzed a single-center cohort of surgically treated colon cancer patients to address three objectives: (i) to describe the incidence of major metabolic and cardiovascular comorbidities and inflammatory biomarkers; (ii) to compare these features across age strata and acute presentation categories (no obstruction, sub-occlusive syndrome, and frank obstruction); and (iii) to explore independent associations between age, metabolic comorbidities, systemic inflammation, and both acute obstruction and length of hospitalization. Direct behavioral exposures (smoking, alcohol, diet) were also captured but sparsely recorded; therefore, we used metabolic comorbidities and biochemical markers as pragmatic, though imperfect, proxies of long-term lifestyle exposure. These indicators are easier to retrieve in retrospective datasets and more consistently available in routine care, but they also reflect tumor-associated catabolism, medication use and acute systemic inflammation.

## 2. Materials and Methods

### 2.1. Study Design and Population

We conducted an observational, single-center cohort study using routinely collected data from adults undergoing colon cancer surgery at the Victor Babes University of Medicine and Pharmacy-affiliated hospitals from Timisoara, Romania. The source dataset comprised 700 consecutive patients admitted to a tertiary surgical unit for histologically confirmed colon or colorectal adenocarcinoma. For the present analysis, we excluded 23 cases due to incomplete data, leading to a sample of 677 patients with complete data on age, core metabolic comorbidities, inflammatory markers, acute presentation, and length of hospital stay.

All patients were managed according to local protocols, with elective or urgent resection performed depending on the presence of obstruction or subocclusive manifestations. The dataset included both emergency and scheduled admissions, reflecting real-world practice where screening-detected lesions, symptomatic but non-obstructing tumors, and acute obstruction coexist.

In this observational cohort, all included patients had histologically confirmed colon cancer and underwent surgical treatment in a tertiary center. The index factors were age, metabolic comorbidity burden (diabetes, hypertension, chronic kidney disease, atrial fibrillation, multimorbidity), and systemic inflammatory/metabolic biomarkers, including C-reactive protein (CRP), D-dimer, fasting glucose, lipid fractions, neutrophil-to-lymphocyte ratio (NLR), platelet-to-lymphocyte ratio (PLR), C-reactive protein-to-albumin ratio (CAR), together with the severity of acute presentation (no obstruction, subocclusive syndrome, frank obstruction). The Comparators were patients with younger age, absent or lower comorbidity counts, more favorable biomarker profiles, and non-obstructive presentation. The primary Outcomes were odds and ordered severity of acute obstructive presentation, while secondary outcomes included length of hospital stay and correlations between inflammatory–metabolic indices and clinical course, analyzed using multivariable regression, correlation, and principal component methods.

### 2.2. Clinical and Lifestyle-Related Variables

Demographic and clinical variables included age (years) and binary indicators of diabetes mellitus, arterial hypertension, chronic obstructive pulmonary disease, asthma, chronic kidney disease (CKD), and atrial fibrillation. These were coded as 1 (present) or 0 (absent) based on medical history. To capture the overall burden of lifestyle-related chronic disease, we constructed a comorbidity count (range 0–6) summing these binary conditions and defined multimorbidity as ≥2 comorbidities. Age was analyzed both continuously and dichotomized as <65 vs. ≥65 years, a conventional threshold in colorectal cancer epidemiology and screening.

Behavioral exposures (smoking, alcohol use, and dietary patterns) were available in the electronic template but were completed for <2% of patients. Given this high degree of missingness, these variables were not included in comparative or multivariable analyses and are interpreted as non-informative in this dataset. Instead, diabetes, hypertension, chronic kidney disease, dyslipidemia, and body-weight-related laboratory indices were considered pragmatic downstream markers of long-term lifestyle exposures relevant to colon carcinogenesis.

Information on admission route (emergency versus elective) was not consistently available across the entire study period, and we therefore did not stratify length-of-stay analyses by this factor. The observed shorter hospital stays in patients with subocclusion or obstruction should thus be interpreted with caution and may partly reflect more streamlined, protocolized pathways for clearly obstructive presentations compared with the more heterogeneous trajectories of non-obstructed patients.

### 2.3. Laboratory Parameters and Outcomes

Preoperative laboratory parameters were extracted from the last routine blood sample obtained before surgery (typically within 24 h before skin incision); in patients undergoing urgent surgery for obstruction, this sample corresponded to the admission laboratory panel. The variables included C-reactive protein (CRP, mg/L), D-dimer (ng/mL), fasting plasma glucose (mg/dL), total cholesterol, LDL cholesterol and HDL cholesterol (all in mg/dL). These markers were chosen as they integrate systemic inflammation, coagulation activation, and cardiometabolic risk, all of which are implicated in colorectal carcinogenesis and may influence the severity of presentation and perioperative course.

Clinical presentation was categorized using two binary fields: subocclusive syndrome (yes/no) and intestinal obstruction (yes/no). From these, we derived a three-level acute presentation variable: 0 = no obstruction (neither subocclusive nor obstructive features), 1 = subocclusive syndrome only, and 2 = frank intestinal obstruction (with or without prior subocclusive symptoms, recognizing that obstruction supersedes subocclusion clinically). A secondary outcome was length of hospital stay, defined as the total number of days from admission to discharge and treated as a continuous variable.

### 2.4. Statistical Analysis

Continuous variables were summarized as mean ± standard deviation (SD) given the large sample size; skewness was inspected visually, but no transformations were applied for primary descriptive summaries. Categorical variables were presented as counts and percentages. Group comparisons for continuous variables between two groups (e.g., <65 vs. ≥65 years; diabetes vs. no diabetes) were performed using the Mann–Whitney U test, chosen for robustness to non-normality. Comparisons across the three acute presentation categories (no obstruction, subocclusive syndrome, obstruction) used the Kruskal–Wallis test. Associations between categorical variables were examined using χ^2^ tests; Fisher’s exact test was reserved for 2 × 2 tables with expected cell counts < 5. Correlations among continuous and ordinal variables (age, comorbidity count, acute presentation coded 0–2, CRP, D-dimer, fasting glucose, lipids, and length of stay) were quantified using Spearman’s rank correlation coefficient (ρ) with two-sided *p*-values.

To explore determinants of acute obstructive presentation, we fitted a multivariable logistic regression model with a binary outcome (any subocclusive/obstructive presentation vs. no obstruction). Predictors included age (per year), comorbidity count, diabetes, hypertension, and CRP entered per 100 mg/L to aid interpretability. To evaluate predictors of length of stay, we fitted a multivariable linear regression with age, acute presentation category, comorbidity count, diabetes, and CRP as covariates. Model estimates are reported as odds ratios (ORs) or regression coefficients (β) with 95% confidence intervals (CIs) and *p*-values. Candidate predictors for each multivariable model were selected a priori based on biological plausibility and previous reports linking age, metabolic comorbidities, diabetes, hypertension and systemic inflammation to colorectal cancer risk and outcomes, while maintaining a conservative events-per-parameter ratio to reduce overfitting. Because inflammatory indices and comorbidity measures can be correlated, we examined pairwise Spearman correlation coefficients among candidate predictors before model fitting and avoided entering strongly collinear variables (pre-specified threshold |ρ| > 0.7) into the same model. In the final models, all correlations between included covariates remained below this threshold, arguing against problematic multicollinearity. Analyses used a complete-case approach for the variables of interest. A two-sided alpha of 0.05 defined statistical significance, and no multiplicity correction was applied.

All analyses were performed using R v4.3.1 (R Foundation for Statistical Computing, Vienna, Austria). Ordinal logistic regression used proportional-odds modeling, and principal component analysis was performed on standardized variables using base R procedures. For the ordinal logistic regression, the proportional odds (parallel slopes) assumption was assessed using standard diagnostics (proportional-odds/parallel-lines testing and inspection of cumulative logit patterns). No material violations were observed; therefore, proportional-odds models are reported.

## 3. Results

Among 677 patients, mean age was 66.6 ± 11.0 years, with 270 (39.9%) younger than 65 years and 407 (60.1%) aged ≥65 years. As expected by design, chronological age differed markedly between groups, but length of hospital stay was remarkably similar (11.6 ± 6.8 vs. 11.5 ± 5.9 days; *p* = 0.398), suggesting that age alone did not drive early inpatient resource use in this cohort. In contrast, lifestyle-related metabolic and cardiovascular comorbidities are clearly concentrated in older patients. Diabetes prevalence almost doubled from 11.9% in those <65 years to 21.4% in those ≥65 years (*p* = 0.002), while hypertension rose from 23.3% to 40.3% (*p* < 0.001). Atrial fibrillation, another marker of chronic cardiovascular remodeling, was more than twice as common in older patients (13.3% vs. 5.2%; *p* = 0.001). Chronic kidney disease was only observed in the older group (1.2%), though absolute numbers were small.

When these conditions were aggregated into a multimorbidity index, 18.4% of older patients versus 9.6% of younger patients had ≥2 comorbidities (*p* = 0.002), underscoring the clustering of metabolic disease later in life. By contrast, the incidence of subocclusive syndrome (≈12% in both groups) and frank intestinal obstruction (21.5% vs. 25.6%; *p* = 0.261) did not significantly differ by age, suggesting that age-related comorbidity accumulation does not straightforwardly translate into a higher probability of presenting with acute obstruction. Inflammatory and lipid profiles were broadly similar, with mean CRP around 200 mg/L and modestly reduced total and LDL cholesterol, consistent with the nutritional and inflammatory milieu typical of cancer patients (Table 1).

Acute presentation patterns were heterogeneous: 441 patients (65.1%) had no obstructive features, 74 (10.9%) presented with subocclusive syndrome, and 162 (23.9%) with frank intestinal obstruction. Age differed modestly but significantly across these categories (*p* = 0.034), with patients in the obstruction group being the oldest on average (68.5 ± 12.2 years) compared with those without obstruction (66.0 ± 10.4 years) and with subocclusive syndrome (65.8 ± 11.6 years). This gradient aligns with the concept that cumulative exposure time to carcinogenic factors and delayed diagnosis in older individuals may predispose them to more advanced luminal compromise. Length of hospital stay showed a counterintuitive pattern: patients without obstruction had the longest mean stay (12.2 ± 6.4 days), whereas those with subocclusive syndrome and obstruction had shorter stays (10.5 ± 5.0 and 10.3 ± 6.1 days, respectively; *p* < 0.001). Several explanations are plausible, including more streamlined diagnostic and surgical pathways in clearly obstructive presentations, earlier postoperative discharge among patients who undergo urgent but definitive resection, or prolonged preoperative staging and optimization among non-obstructed patients. Notably, the prevalence of diabetes (19.7%, 16.2%, and 12.3% across no obstruction, subocclusive, and obstruction groups, respectively; *p* = 0.102) and hypertension (32.7%, 29.7%, 37.7%; *p* = 0.393) did not differ significantly, nor did the proportion with ≥2 comorbidities (~13–15% across groups). CRP values were uniformly high (≈180–203 mg/L) without meaningful variation (*p* = 0.315), as seen in Table 2.

A total of 119 patients (17.6%) had diabetes. Patients with diabetes were older than those without diabetes (69.5 ± 7.7 vs. 66.0 ± 11.5 years; *p* = 0.007). Fasting glucose values were substantially higher in the diabetes group (182.6 ± 69.3 vs. 157.4 ± 73.9 mg/dL; *p* < 0.001), validating the historical diagnosis and reflecting a mix of chronic dysglycemia and stress hyperglycemia. Interestingly, CRP tended to be lower in patients with diabetes (183.3 ± 110.9 vs. 203.7 ± 113.3 mg/L), though the difference did not reach conventional statistical significance (*p* = 0.071), and D-dimer levels were slightly higher (8.7 ± 4.1 vs. 8.0 ± 4.2 ng/mL, *p* = 0.099). Lipid profiles were broadly similar, with a weak, non-significant trend toward higher LDL cholesterol in diabetics (88.6 ± 32.5 vs. 83.1 ± 31.7 mg/dL; *p* = 0.089), while HDL cholesterol was almost identical. Length of stay did not differ meaningfully (11.9 ± 5.5 vs. 11.5 ± 6.4 days; *p* = 0.146), suggesting that, within this surgical context, diabetes per se did not prolong hospitalization, although unmeasured factors (e.g., perioperative glycemic control, complications) may play a role. Of note, the proportion experiencing frank intestinal obstruction was numerically lower among diabetics (16.8% vs. 25.4%; *p* = 0.059), a borderline finding that may be due to chance, sample size, or more intensive medical surveillance in diabetic patients leading to earlier detection before obstructive complications (Table 3 and Table 4).

Age correlated positively with comorbidity count (ρ = 0.265; *p* < 0.001), confirming that older colon cancer patients accumulate more lifestyle-related chronic diseases such as diabetes, hypertension, and chronic kidney disease. Age also showed a small but statistically significant positive association with the ordinal acute presentation scale (0 = no obstruction, 2 = frank obstruction; ρ = 0.088; *p* = 0.022), echoing the groupwise findings in Table 1 and Table 2 and supporting the notion that older individuals are modestly more likely to present with obstructive phenomena. Length of stay correlated weakly but significantly with both fasting glucose (ρ = 0.082; *p* = 0.032) and LDL cholesterol (ρ = 0.080; *p* = 0.039), suggesting that patients with poorer metabolic health may experience slightly longer hospitalizations, although effect sizes are small. Interestingly, length of stay was negatively correlated with the severity of acute presentation (ρ = −0.179; *p* < 0.001), reinforcing the earlier observation that patients without obstruction tended to stay longer, possibly because their care pathways involve more diagnostic staging, adjuvant planning, or management of comorbidities. Fasting glucose displayed a negative correlation with both LDL cholesterol (ρ = −0.081; *p* = 0.036) and acute presentation severity (ρ = −0.135; *p* < 0.001), indicating that patients with higher glucose levels tended to have lower LDL values and less obstructive presentation; this pattern might reflect complex interactions among nutritional status, catabolic stress, and chronic medication use (e.g., statins), but causality cannot be inferred. Finally, LDL cholesterol correlated positively with comorbidity count (ρ = 0.103; *p* = 0.007), consistent with its role as a component of metabolic syndrome (Table 5).

The multivariable logistic regression model explored whether age, cumulative comorbidity burden, diabetes, hypertension, and systemic inflammation independently predicted an acute obstructive presentation. After adjustment for comorbidity count, diabetes, hypertension and CRP, age emerged as the only statistically significant host-related predictor of acute obstructive presentation in our models: each additional year of age was associated with a 1.6% increase in the odds of presenting with subocclusive syndrome or frank obstruction (OR 1.016; 95% CI 1.001–1.032; *p* = 0.038). In contrast, the comorbidity count did not meaningfully influence the odds of obstruction (OR 0.927; 95% CI 0.585–1.471; *p* = 0.749), suggesting that, in this dataset, having multiple lifestyle-related chronic diseases did not increase the likelihood of presenting emergently once age was accounted for. Similarly, diabetes was associated with a non-significant trend toward lower odds of acute obstruction (OR 0.630; 95% CI 0.325–1.222; *p* = 0.171), echoing the descriptive finding of fewer obstructive events among diabetics; this may reflect closer surveillance, earlier symptom recognition, or residual confounding. Hypertension showed no clear effect (OR 1.205; 95% CI 0.677–2.145; *p* = 0.526). CRP entered per 100 mg/L, a proxy for systemic inflammation, was not independently associated with acute obstruction (OR 0.932; 95% CI 0.809–1.074; *p* = 0.329), as seen in Table 6. We did not adjust this model for tumor location or stage, which were not available in standardized form in our database and therefore could not be reliably incorporated.

In the multivariable linear regression model, length of hospital stay was modeled as a function of age, acute presentation severity, comorbidity burden, diabetes, and CRP. Strikingly, acute presentation category emerged as the only independent predictor: each one-step increase on the 0–2 scale (from no obstruction to subocclusive syndrome to frank obstruction) was associated with a nearly one-day reduction in hospital stay (β = −0.959 days; 95% CI −1.517 to −0.402; *p* = 0.001). Thus, compared with non-obstructed patients, those with obstruction were predicted to stay roughly two days less, after adjusting for age, comorbidities, diabetes, and CRP. This counterintuitive finding likely reflects structural differences in care pathways: obstructed patients may undergo expedited work-up, proceed rapidly to surgery, and have fewer delays related to diagnostic staging or adjuvant planning, whereas non-obstructed patients may experience longer preoperative evaluation or more complex postoperative management for comorbid conditions. Age was not significantly associated with length of stay after adjustment (β = −0.015 days per year; *p* = 0.508), and neither was comorbidity count (β = 0.654; *p* = 0.116), though the point estimate suggests a trend toward longer stay with higher comorbidity burden that the current sample may be underpowered to confirm. Diabetes had no independent effect (β = −0.541; *p* = 0.511), consistent with the descriptive analyses. CRP did not predict length of stay (β ≈ 0 per mg/L; *p* = 0.672), as presented in Table 7.

Spearman correlation analysis (Table 8) demonstrated a moderate positive association between age and comorbidity burden (ρ = 0.265, *p* < 0.0001), confirming that multimorbidity clusters in older colon cancer patients. By contrast, age showed no relevant correlation with neutrophil-to-lymphocyte ratio (NLR), platelet-to-lymphocyte ratio (PLR), or CRP/albumin ratio (CAR), and was not associated with length of stay, indicating that the inflammatory and composite immune–nutritional indices were largely age-independent in this cohort. The only strong correlation observed was between NLR and PLR (ρ = 0.530, *p* < 0.0001), reflecting their shared construction from lymphocyte counts and underscoring that they partly capture overlapping immunologic information. Neither NLR nor PLR nor CAR showed meaningful correlation with length of stay.

Principal component analysis (Table 9) was used to reduce the joint inflammatory–metabolic profile (NLR, CAR, fasting glucose, total cholesterol, LDL, HDL) into latent dimensions. PC1, explaining 18.8% of variance, loaded positively on CAR (0.539), LDL (0.443), total cholesterol (0.377), and NLR (0.387), and negatively on glucose (−0.448), capturing a mixed “dyslipidemia–inflammatory” axis where higher LDL, systemic inflammation, and relative lymphopenia co-occur. PC2 (18.3% of variance) contrasted NLR (0.609) and CAR (0.373) against LDL (−0.622) and total cholesterol (−0.302), suggesting a spectrum from lipid-dominant to inflammation-dominant phenotypes at similar glycemic levels. PC3 (17.4% of variance) was driven almost entirely by HDL cholesterol (loading 0.877), delineating an orthogonal “HDL-rich” axis that is statistically independent of the other components and provides a more comprehensive view of lifestyle-related systemic milieu in colon cancer than single biomarkers alone.

All variables were standardized before PCA. We retained three components based on eigenvalues > 1 and visual inspection of the scree plot. PC1, PC2 and PC3 explained 18.8%, 18.3% and 17.4% of the total variance, respectively (cumulative 54.5%), indicating that a substantial proportion of biomarker variability remains unexplained and that these components should be interpreted as broad, low-resolution summaries of the inflammatory–metabolic milieu rather than as definitive phenotypes.

An ordinal logistic regression (Table 10) modeled the ordered severity of acute presentation (none → subocclusive → frank obstruction) as a function of age, immune–inflammatory indices, and metabolic comorbidities. Age emerged as a significant predictor: each additional year was associated with a 1.8% increase in the odds of being in a more severe acute category (OR 1.018; 95% CI 1.003–1.033; *p* = 0.017), supporting an exposure-time effect whereby older patients are slightly more likely to present with luminal compromise. NLR showed a borderline association (OR 1.013 per unit; 95% CI 0.998–1.029; *p* = 0.090), suggesting that a more pro-inflammatory leukocyte profile may modestly favor obstructive presentation, although the confidence interval includes the null. CAR and hypertension were not significantly associated with acute severity. Interestingly, diabetes was inversely associated with more severe presentation (OR 0.573; 95% CI 0.368–0.893; *p* = 0.0139).

**Figure 1:**
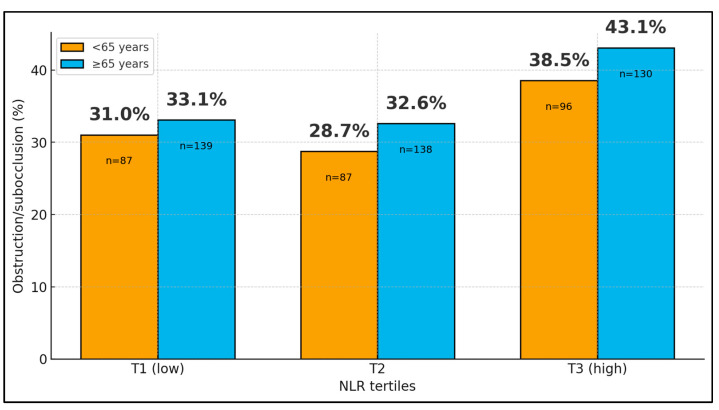
Acute obstructive presentation by age group and NLR tertiles.

**Figure 2:**
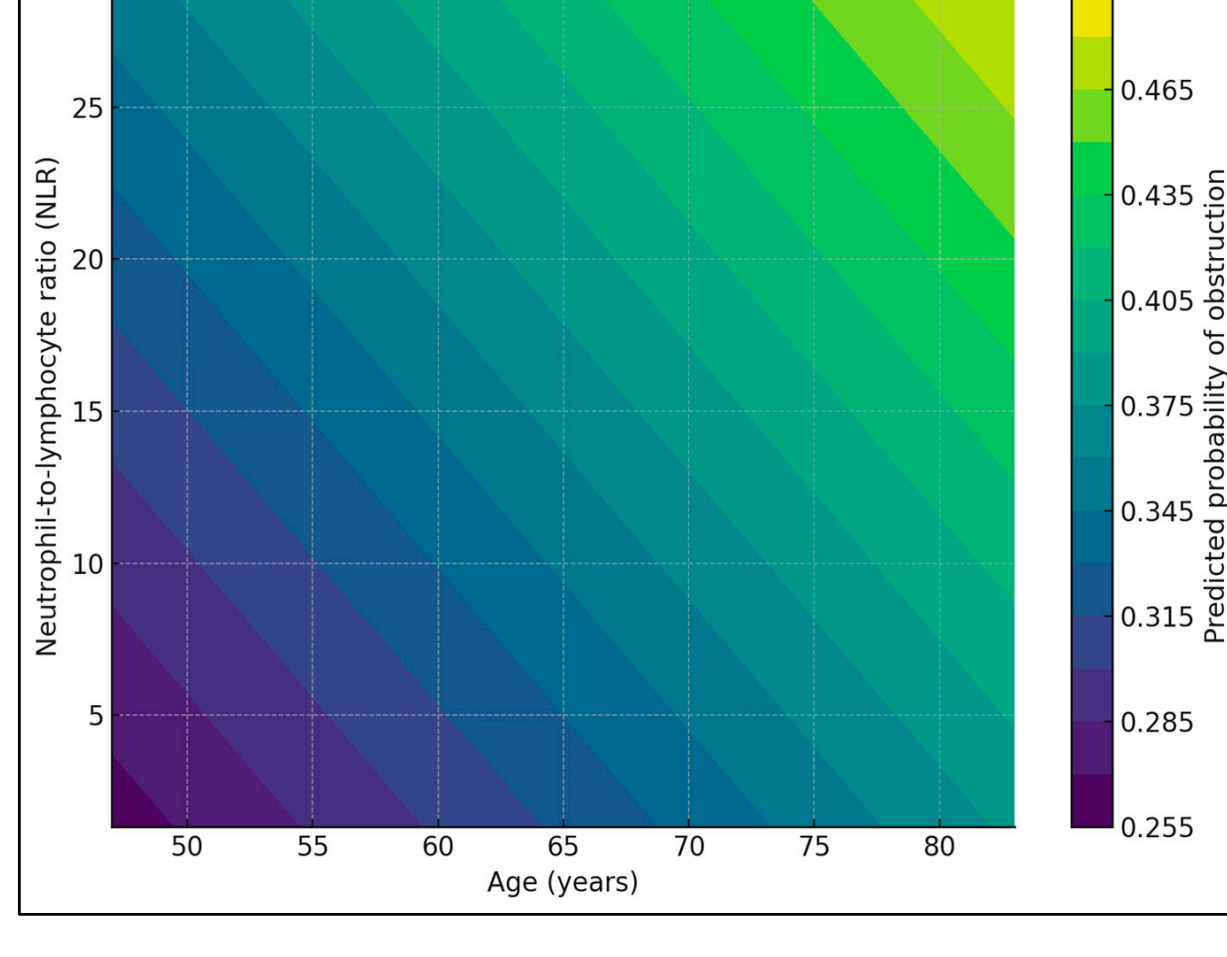
Predicted probability of obstructive presentation pattern by NLR.

When NLR was categorized into tertiles, the overall proportion of patients with subocclusive or obstructive presentation increased from 32.3% in the lowest tertile (T1, 73/226) to 31.1% in the middle tertile (T2, 70/225) and 41.2% in the highest tertile (T3, 93/226). Stratification by age group showed similar gradients in both younger and older patients: among those <65 years, obstruction rates were 31.0% (27/87), 28.7% (25/87), and 38.5% (37/96) across T1, T2, and T3, respectively; in patients ≥ 65 years, the corresponding rates were 33.1% (46/139), 32.6% (45/138), and 43.1% (56/130). In a multivariable logistic model adjusting for age group, belonging to the highest NLR tertile versus the lowest was associated with a 47% increase in the odds of obstructive presentation (OR 1.48; 95% CI 1.00–2.17; *p* = 0.048), whereas age group itself was not a significant predictor (*p* = 0.35), as seen in Figure 1 and Figure 2.

Between 2013 and 2023, the institutional caseload ranged from 38 to 80 colon cancer surgeries per year, with mean age consistently around 65–68 years (Table 11). The proportion of early-onset cases (<50 years) fluctuated between ~3% and ~21% without a clear linear trend (logistic regression with year as a continuous predictor: OR 0.94 per year; 95% CI 0.87–1.03; *p* = 0.20). The proportion of patients presenting with subocclusive syndrome or frank obstruction varied by calendar year (~23% to ~58%); modeling obstruction (yes/no) against year suggested a modest decrease in the odds of obstructive presentation over time (OR 0.95 per year; 95% CI 0.90–1.00; *p* = 0.044).

**Figure 3:**
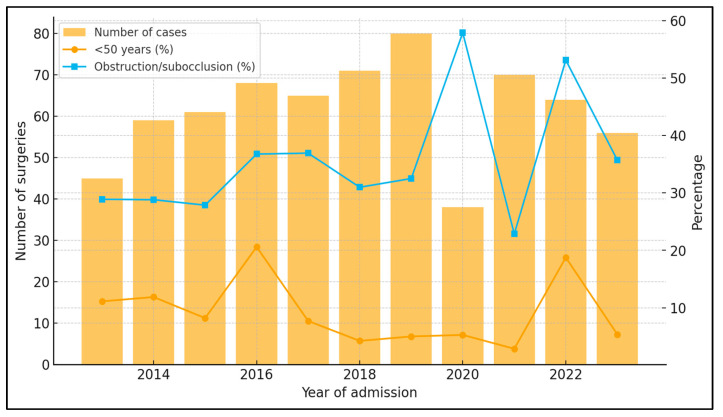
Annual caseload, early-onset cases, and obstructive presentation (2013–2023).

Between 2013 and 2023, the annual surgical caseload ranged from 38 to 80 patients, with a median of 65 cases/year. The proportion of early-onset colon cancer (<50 years) fluctuated between 2.9% (2021) and 20.6% (2016), with no clear linear trend: logistic regression of early-onset status on calendar year yielded an odds ratio (OR) of 0.94 per year (95% CI 0.87–1.03; *p* = 0.20), corresponding to a modeled probability of 11.7% in 2013 versus 7.0% in 2023. In contrast, the percentage of patients presenting with subocclusive syndrome or frank obstruction varied between 22.9% (2021) and 57.9% (2020), as seen in Figure 3.

Logistic regression showed a modest but significant decrease in the odds of obstructive presentation over time (OR 0.95 per year; 95% CI 0.90–1.00; *p* = 0.044). Points show observed annual proportions, and the solid line depicts model-fitted probabilities over time (with 95% confidence bands).

## 4. Discussion

### 4.1. Analysis of Findings

In this cohort, we found a clear age gradient in multimorbidity, with diabetes, hypertension and atrial fibrillation clustering in older adults, but only a modest association between age and obstructive presentation and no independent effect of comorbidity count. This pattern partly diverges from population-level data, where metabolic syndrome and its components are consistently associated with higher CRC incidence and earlier onset, especially when hyperglycemia and central obesity coexist. Large cohort analyses confirm that metabolic syndrome, hyperglycemia and related metabolic traits increase CRC risk and contribute to earlier-onset disease, particularly in the proximal colon [16,17,18].

Our age–multimorbidity correlation therefore likely reflects the accumulation of lifestyle-related damage over time, whereas the weak translation into obstructive presentation suggests that once cancer has developed, factors beyond traditional cardiometabolic comorbidities—such as tumor location, growth pattern and health-system pathways—become dominant determinants of how patients present. At the same time, in a surgical cancer population, these cardiometabolic comorbidities and biochemical markers represent composite phenotypes shaped not only by long-term behavioral exposures but also by tumor-associated catabolism, systemic inflammation and prior medical treatment, and they should not be interpreted as pure surrogates of lifetime lifestyle risk.

The behavior of diabetes in our dataset illustrates this complexity. Although diabetic patients were older and had markedly higher fasting glucose, they were not more likely to present with obstruction and showed no excess length of stay. This contrasts with the strong link between diabetes/metabolic syndrome and CRC incidence reported in observational and genetic-epidemiology studies, where metabolic syndrome and its components increase CRC risk by roughly 20–30% and appear to predispose to earlier-onset disease and more proximal tumors [17,18]. At the same time, work in surgically treated cohorts suggests that metabolic syndrome is less prevalent in very advanced CRC, possibly because cancer-related weight loss and catabolism obscure classical metabolic features [16].

Our finding of similar or even lower obstruction rates in diabetics may reflect closer longitudinal follow-up and more opportunities for symptom recognition in this group, rather than a genuinely less aggressive tumor biology, and underlines the need to disentangle biological from surveillance-driven effects in future studies.

Our null results for NLR, PLR and CAR with respect to age, multimorbidity and length of stay also contrast with a rapidly growing literature that positions these indices as robust prognostic markers in CRC. In a Romanian colon cancer cohort, higher systemic NLR was independently associated with advanced stage and poorer overall survival [19], and updated meta-analyses have confirmed that elevated CAR carries adverse prognostic information across colorectal cancer stages [20]. Mungan et al. further showed that a high C-reactive protein–lymphocyte ratio predicts in-hospital mortality after colorectal surgery [21], while a recent prospective MDPI study in colon cancer linked higher preoperative CAR with larger tumor volume, more advanced T and N stages and worse survival [22,23,24,25,26]. One likely explanation is that in our retrospective dataset, these indices were measured at a single preoperative time point that may capture variable mixtures of chronic low-grade inflammation and acute physiological derangement related to obstruction, infection or recent interventions. Furthermore, we focused on short-term outcomes (obstructive presentation and early length of stay) rather than on postoperative complications or long-term survival, which are the endpoints most consistently associated with NLR, PLR and CAR in the literature. Differences in sampling windows, outcome definitions and adjustment for tumor stage may therefore attenuate the apparent prognostic value of these indices in our models.

Beyond NLR, PLR and CAR, several composite inflammatory–nutritional indices such as the prognostic nutritional index (PNI), systemic immune–inflammation index (SII), controlling nutritional status (CONUT) score and the monocyte-to-HDL-cholesterol ratio have shown prognostic value for stage, postoperative morbidity and survival in colorectal cancer cohorts. Likewise, liver- and steatosis-related scores that combine aminotransferases, platelets, lipids and anthropometric measures (AAR, APRI, AARPRI, FIB-4 and the fatty liver index) are increasingly used to capture metabolic liver disease and have been associated with outcomes in both gastrointestinal and extra-gastrointestinal malignancies. However, our institutional database did not systematically record several key components required to calculate these indices (for example, AST, ALT, γ-glutamyl transferase, triglycerides and body mass index), so we were unable to derive them reliably in this cohort. The restricted inflammatory and nutritional panel that we were able to assemble from routine preoperative blood tests likely contributes to the modest discriminative performance observed for NLR, PLR and CAR with respect to obstructive presentation and early in-hospital course. However, because these three components captured only approximately 55% of the total variance, their clinical interpretability is limited, and they should be viewed as exploratory descriptive tools rather than as ready-to-use risk scores.

In our cohort, by contrast, NLR, PLR and CAR were largely age-independent and did not correlate with length of stay, suggesting that these markers may be more informative for long-term oncologic and complication-related outcomes than for the short-term resource use that we analyzed. Similarly, the modest explanatory value of NLR and PLR for acute presentation in our models stands in contrast to evidence from prognostic cohorts. Kim et al. reported that both NLR and PLR independently predicted poorer overall and progression-free survival in stage III–IV colorectal cancer [22], and Josse et al. found that an elevated NLR was associated with major perioperative complications in patients undergoing colorectal surgery [23].

In our series, NLR and PLR were strongly correlated with each other (as expected from shared leukocyte denominators) but did not track with obstruction severity or length of stay. These differences likely reflect outcome choice (complications and survival versus length of stay), timing of biomarker measurement (standardized preoperative sampling versus heterogeneous perioperative windows) and the absence of detailed staging and postoperative complication data in our dataset, all of which can attenuate the discriminative performance of inflammatory scores.

When we examined time trends, we observed a modest decline in the odds of obstructive presentation over the 2013–2023 period, albeit with substantial year-to-year variability. This pattern is directionally consistent with population-based data from Canada, where the introduction of organized screening was followed by a marked reduction in CRC diagnoses with obstruction, perforation and emergency admission [24]. A further methodological consideration is potential collider bias. Inclusion in our cohort required both a diagnosis of colon cancer and suitability for surgical treatment in a tertiary center. Patients with a heavy burden of cardiometabolic comorbidities may have had more frequent contact with healthcare services and thus more opportunities for earlier symptom recognition, lowering their probability of presenting with frank obstruction. Conversely, individuals with both severe comorbidities and very advanced or unresectable cancers may have been channeled towards non-surgical or palliative pathways and are therefore absent from our dataset. This pattern is directionally consistent with population-based data showing reductions in obstructive or emergency colorectal cancer diagnoses after the introduction of organized screening programs.

However, unlike large registries, our single-center series cannot fully capture changes in screening uptake, referral behavior or stage migration. Large European studies have shown that patients with multiple comorbidities are less likely to be screen-detected and more likely to receive an emergency diagnosis, with higher short-term mortality [25,26].

Overall, our findings highlight the complex and sometimes counterintuitive phenotype of surgically treated colon cancer at the interface of lifestyle exposure, metabolic health, systemic inflammation and health-system factors. The strong age–multimorbidity correlation and high prevalence of metabolic comorbidities in our cohort align with current reviews emphasizing obesity and metabolic syndrome as central drivers of CRC incidence and earlier-onset disease and calling for tailored screening strategies in metabolically high-risk populations [17,18].

Yet, acute obstructive presentation and early length of stay appeared to be driven mainly by age and organizational factors, with only modest contributions from metabolic and inflammatory markers. In line with registry studies on diagnostic routes, this suggests that social determinants, access to endoscopy, and primary–secondary care integration may be as important as biological risk in shaping how and when patients reach surgery [24,25,26]. Future work combining detailed staging, molecular profiling, standardized inflammatory indices and data on socioeconomic status and care pathways will be needed to build integrated risk models that can both identify metabolically vulnerable individuals before cancer develops and reduce the likelihood of emergency presentation among those who do.

These findings suggest that in colon cancer, old age is a more consistent determinant of obstructive presentation than the mere accumulation of lifestyle-related comorbidities or elevated inflammatory markers. Our interpretation of these components follows standard guidance on the use of principal component analysis for dimensionality reduction in biomedical datasets [27]. Clinicians should therefore maintain a high index of suspicion for subocclusive or obstructive symptoms in older adults, irrespective of their formal multimorbidity burden and not assume that patients with diabetes or hypertension necessarily have a higher likelihood of emergency presentation. The inverse association between obstruction severity and length of stay indicates that streamlined diagnostic and surgical pathways for clearly obstructive cases may shorten hospitalizations, whereas non-obstructed patients may require more protracted pre- and postoperative management. From a prevention standpoint, the frequent coexistence of colon cancer with metabolic derangements reinforces the need for integrated cardiometabolic and cancer-preventive strategies in primary care but also highlights that short-term surgical outcomes cannot be reliably risk-stratified using simple inflammatory or lipid indices alone. Nevertheless, these findings should be interpreted in light of potential residual confounding from unmeasured or incompletely controlled factors, including underlying comorbidities and other patient- and treatment-related characteristics [28,29,30,31,32,33].

### 4.2. Study Limitations

This single-center, observational analysis of routinely collected data is inherently vulnerable to residual confounding and selection bias. In particular, the cohort consists exclusively of patients who underwent surgical resection in a tertiary unit; individuals with unresectable disease, severe frailty or major comorbid constraints, and those treated non-operatively or with palliative intent are not represented. Detailed behavioral exposures (diet, physical activity, smoking, alcohol) were sparsely recorded and had to be proxied by metabolic comorbidities and biochemical markers, which may incompletely capture lifetime lifestyle risk. Tumor-related variables (pathological stage, sidedness and histologic subtype) and perioperative complications were not integrated into the multivariable models because they were not captured in a standardized fashion in the electronic database and were missing for a substantial minority of patients. This limits mechanistic interpretation of the links between obstruction, biomarkers and length of stay and may partly explain the modest discriminative performance of the host-related indices we examined. As a result, the metabolic comorbidities and lipid/inflammatory markers that we used as lifestyle proxies integrate both long-term exposure and short-term effects of cancer cachexia, malnutrition and acute-phase responses, and they cannot be interpreted as pure measures of lifetime behavioral risk. Immune–inflammatory indices (NLR, PLR, CAR) were measured at a single preoperative time point and may reflect acute stress rather than stable phenotypes. Moreover, we could not compute a broader spectrum of non-invasive inflammatory and hepatic steatosis scores (including PNI, SII, CONUT, monocyte-to-HDL ratio, AAR, APRI, AARPRI, FIB-4 and the fatty liver index), because several of their required components were either not captured at all or were available only in a minority of patients. Our analyses may therefore underestimate the prognostic information contained in the systemic inflammatory–nutritional milieu compared with studies that prospectively collect these variables. Finally, the exploratory nature of multiple comparisons without adjustment increases the risk of chance findings, and external validity to other institutions and health systems remains to be established.

## 5. Conclusions

In a large hospital-based cohort of surgically treated colon cancer patients, aging was tightly linked to the accumulation of metabolic comorbidities but emerged as the only consistent predictor of obstructive presentation, while multimorbidity and systemic inflammatory markers showed limited discriminatory value for either acute obstruction or early in-hospital resource use. Acute presentation category, paradoxically associated with shorter length of stay, appears to capture differences in care pathways rather than purely disease severity. Composite inflammatory–metabolic dimensions derived from principal component analysis summarized biomarker variance but did not translate into clear gradients in obstruction or hospitalization. Future work integrating detailed tumor biology, longitudinal lifestyle data, and post-discharge outcomes is needed to refine risk stratification and to clarify how metabolic health and systemic inflammation shape both the clinical presentation and trajectory of colon cancer. Because our analyses are restricted to surgically treated patients in a single tertiary center and our models did not adjust for tumor location, growth pattern or stage, these findings should be interpreted as hypothesis-generating and are not directly generalizable to non-surgical or population-based colon cancer cohorts. Future work integrating detailed tumor characteristics with comprehensive metabolic and inflammatory profiling in broader patient populations will be needed to refine risk stratification for obstructive presentation and early in-hospital course.

## Figures and Tables

**Table 1:** Baseline characteristics overall and by age group (<65 vs. ≥65 years).

Variable	Overall (n = 677)	<65 Years (n = 270)	≥65 Years (n = 407)	*p*-Value *
Age, years (mean ± SD)	66.6 ± 11.0	55.7 ± 7.4	73.8 ± 6.0	—
Length of stay, days (mean ± SD)	11.5 ± 6.3	11.6 ± 6.8	11.5 ± 5.9	0.398
Diabetes, n (%)	119 (17.6)	32 (11.9)	87 (21.4)	0.002
Hypertension, n (%)	227 (33.5)	63 (23.3)	164 (40.3)	<0.001
COPD, n (%)	1 (0.1)	1 (0.4)	0 (0.0)	0.399
Asthma, n (%)	14 (2.1)	3 (1.1)	11 (2.7)	0.179
Chronic kidney disease, n (%)	5 (0.7)	0 (0.0)	5 (1.2)	0.163
Atrial fibrillation, n (%)	68 (10.0)	14 (5.2)	54 (13.3)	0.001
≥2 comorbidities, n (%)	101 (14.9)	26 (9.6)	75 (18.4)	0.002
Subocclusive syndrome, n (%)	83 (12.3)	34 (12.6)	49 (12.0)	0.924
Intestinal obstruction, n (%)	162 (23.9)	58 (21.5)	104 (25.6)	0.261
CRP, mg/L (mean ± SD)	200.1 ± 113.1	202.6 ± 114.3	198.4 ± 112.4	0.606
Total cholesterol, mg/dL (mean ± SD)	94.7 ± 40.0	97.2 ± 39.5	93.1 ± 40.2	0.186
LDL cholesterol, mg/dL (mean ± SD)	84.0 ± 31.9	82.7 ± 30.6	84.9 ± 32.7	0.39
HDL cholesterol, mg/dL (mean ± SD)	54.8 ± 18.9	54.7 ± 18.9	54.9 ± 18.9	0.896

* Mann–Whitney U test for continuous variables; χ^2^ or Fisher’s exact test for categorical variables; Abbreviations: SD, standard deviation; COPD, chronic obstructive pulmonary disease; CRP, C-reactive protein; LDL, low-density lipoprotein cholesterol; HDL, high-density lipoprotein cholesterol; n, number.

**Table 2:** Clinical characteristics by acute presentation (no obstruction, subocclusive syndrome, intestinal obstruction).

Variable	Overall (n = 677)	No Obstruction (n = 441)	Subocclusive (n = 74)	Obstruction (n = 162)	*p*-Value
Age, years (mean ± SD)	66.6 ± 11.0	66.0 ± 10.4	65.8 ± 11.6	68.5 ± 12.2	0.034
Length of stay, days (mean ± SD)	11.5 ± 6.3	12.2 ± 6.4	10.5 ± 5.0	10.3 ± 6.1	<0.001
Diabetes, n (%)	119 (17.6)	87 (19.7)	12 (16.2)	20 (12.3)	0.102
Hypertension, n (%)	227 (33.5)	144 (32.7)	22 (29.7)	61 (37.7)	0.393
≥2 comorbidities, n (%)	101 (14.9)	66 (15.0)	10 (13.5)	25 (15.4)	0.928

Kruskal–Wallis test for continuous variables; χ^2^ test for categorical variables; Abbreviations: SD, standard deviation; n, number.

**Table 3:** Inflammatory and metabolic biomarkers in patients with and without diabetes.

Variable	Overall (n = 677)	No Diabetes (n = 558)	Diabetes (n = 119)	*p*-Value *
Age, years (mean ± SD)	66.6 ± 11.0	66.0 ± 11.5	69.5 ± 7.7	0.007
Length of stay, days (mean ± SD)	11.5 ± 6.3	11.5 ± 6.4	11.9 ± 5.5	0.146
CRP, mg/L (mean ± SD)	200.1 ± 113.1	203.7 ± 113.3	183.3 ± 110.9	0.071
D-dimer, ng/mL (mean ± SD)	8.2 ± 4.1	8.0 ± 4.2	8.7 ± 4.1	0.099
Fasting glucose, mg/dL (mean ± SD)	161.8 ± 73.7	157.4 ± 73.9	182.6 ± 69.3	<0.001
Total cholesterol, mg/dL (mean ± SD)	94.7 ± 40.0	94.8 ± 39.8	94.4 ± 40.7	0.946
LDL cholesterol, mg/dL (mean ± SD)	84.0 ± 31.9	83.1 ± 31.7	88.6 ± 32.5	0.089
HDL cholesterol, mg/dL (mean ± SD)	54.8 ± 18.9	54.7 ± 19.0	55.2 ± 18.3	0.801
Intestinal obstruction, n (%)	162 (23.9)	142 (25.4)	20 (16.8)	0.059

* Mann–Whitney U test for continuous variables; χ^2^ test for categorical variables; Abbreviations: SD, standard deviation; CRP, C-reactive protein; D-dimer, fibrin degradation product D-dimer; LDL, low-density lipoprotein cholesterol; HDL, high-density lipoprotein cholesterol; n, number.

**Table 4:** Preoperative inflammatory and lipid markers overall and by age group (<65 vs. ≥65 years).

Variable	Overall (n = 677)	<65 Years (n = 270)	≥65 Years (n = 407)	*p*-Value
CRP, mg/L (mean ± SD)	200.1 ± 113.1	202.6 ± 114.3	198.4 ± 112.4	0.606
Total cholesterol, mg/dL	94.7 ± 40.0	97.2 ± 39.5	93.1 ± 40.2	0.186
LDL cholesterol, mg/dL	84.0 ± 31.9	82.7 ± 30.6	84.9 ± 32.7	0.390
HDL cholesterol, mg/dL	54.8 ± 18.9	54.7 ± 18.9	54.9 ± 18.9	0.896

Mann–Whitney U-test; Abbreviations: SD, standard deviation; CRP, C-reactive protein; LDL, low-density lipoprotein cholesterol; HDL, high-density lipoprotein cholesterol; n, number.

**Table 5:** Significant Spearman correlations among key variables.

Variable 1	Variable 2	Spearman ρ	*p*-Value
Age	Comorbidity count	0.265	<0.001
Age	Acute presentation (0–2)	0.088	0.022
Length of stay	Glucose	0.082	0.032
Length of stay	LDL cholesterol	0.08	0.039
Length of stay	Acute presentation (0–2)	−0.179	<0.001
Glucose	LDL cholesterol	−0.081	0.036
Glucose	Acute presentation (0–2)	−0.135	<0.001
LDL cholesterol	Comorbidity count	0.103	0.007

Outcome: three-level acute presentation variable: 0 = no obstruction (neither subocclusive nor obstructive features), 1 = subocclusive syndrome only, and 2 = frank intestinal obstruction (with or without prior subocclusive symptoms, recognizing that obstruction supersedes subocclusion clinically).; Abbreviations: LDL, low-density lipoprotein cholesterol; ρ, Spearman’s rank correlation coefficient.

**Table 6:** Multivariable logistic regression for any acute obstructive presentation (subocclusive or obstructive vs. none).

Predictor	OR	95% CI	*p*-Value
Age, per year	1.016	1.001–1.032	0.038
Comorbidity count, per unit	0.927	0.585–1.471	0.749
Diabetes	0.63	0.325–1.222	0.171
Hypertension	1.205	0.677–2.145	0.526
CRP, per 100 mg/L	0.932	0.809–1.074	0.329

Outcome: acute presentation. Abbreviations: OR, odds ratio; CI, confidence interval; CRP, C-reactive protein.

**Table 7:** Multivariable linear regression for length of hospital stay.

Predictor	β (Days)	95% CI	*p*-Value
Age, per year	−0.015	−0.059 to 0.029	0.508
Acute presentation (0–2)	−0.959	−1.517 to −0.402	0.001
Comorbidity count	0.654	−0.162 to 1.470	0.116
Diabetes	−0.541	−2.155 to 1.073	0.511
CRP, per 1 mg/L	0.001	−0.003 to 0.005	0.672

Abbreviations: β, regression coefficient; CI, confidence interval; CRP, C-reactive protein.

**Table 8:** Spearman correlations between age, multimorbidity, immune–inflammatory indices, and length of stay.

Variable 1	Variable 2	Spearman ρ	*p*-Value
age, years	Comorbidity count (0–4)	0.265	<0.0001
age	NLR	−0.026	0.4963
age	PLR	−0.016	0.6701
age	CAR	−0.052	0.1802
age	Length of stay (days)	0.025	0.5198
Comorbidity count	NLR	0.005	0.8926
Comorbidity count	PLR	0.003	0.9455
Comorbidity count	CAR	−0.022	0.5667
Comorbidity count	Length of stay (days)	0.055	0.1512
NLR (neutrophil/lymph)	PLR (platelet/lymph)	0.53	<0.0001
NLR	CAR (CRP/albumin)	0.034	0.3768
NLR	Length of stay (days)	0.058	0.1329
PLR	CAR	0.019	0.6173
PLR	Length of stay (days)	−0.014	0.7144
CAR	Length of stay (days)	−0.005	0.8899

Abbreviations: NLR, neutrophil-to-lymphocyte ratio; PLR, platelet-to-lymphocyte ratio; CAR, C-reactive protein-to-albumin ratio; ρ, Spearman’s rank correlation coefficient.

**Table 9:** Principal component analysis of inflammatory and metabolic variables.

Variable	PC1 Loading	PC2 Loading	PC3 Loading
NLR	0.387	0.609	0.191
CAR	0.539	0.373	−0.094
Glycemia (glucose)	−0.448	0.082	0.325
Cholesterol total	0.377	−0.302	−0.218
LDL cholesterol	0.443	−0.622	0.178
HDL cholesterol	0.144	−0.072	0.877

All variables were standardized (z-scores) prior to PCA. PC1, PC2, and PC3 explained 18.8%, 18.3%, and 17.4% of total variance, respectively (cumulative 54.5%); Abbreviations: NLR, neutrophil-to-lymphocyte ratio; CAR, C-reactive protein-to-albumin ratio; LDL, low-density lipoprotein cholesterol; HDL, high-density lipoprotein cholesterol; PC, principal component.

**Table 10:** Ordinal logistic regression for severity of acute presentation.

Predictor	OR (per Unit)	95% CI	*p*-Value
age (per year)	1.018	1.003–1.033	0.0171
NLR (per 1 unit)	1.013	0.998–1.029	0.0902
CAR (per 1 unit)	1.014	0.980–1.049	0.4271
Diabetes (yes vs. no)	0.573	0.368–0.893	0.0139
Hypertension (yes/no)	1.165	0.831–1.635	0.3751

Outcome: acute presentation coded as an ordered outcome; 0 = no obstruction, 1 = subocclusive syndrome only, 2 = frank intestinal obstruction; Proportional odds (cumulative logit) model; OR > 1 indicates higher odds of being in a more severe acute presentation category (subocclusive or obstructive); Abbreviations: NLR, neutrophil-to-lymphocyte ratio; CAR, C-reactive protein-to-albumin ratio; OR, odds ratio; CI, confidence interval.

**Table 11:** Annual case load, age, early-onset proportion, and obstructive presentation (2013–2023).

Year	N Cases	Mean Age, Years	Early-Onset < 50, n (%)	Obstruction/Subocclusion, n (%)
2013	45	67.6	5 (11.1%)	13 (28.9%)
2014	59	65.1	7 (11.9%)	17 (28.8%)
2015	61	66	5 (8.2%)	17 (27.9%)
2016	68	62.1	14 (20.6%)	25 (36.8%)
2017	65	67.6	5 (7.7%)	24 (36.9%)
2018	71	68.5	3 (4.2%)	22 (31.0%)
2019	80	67.8	4 (5.0%)	26 (32.5%)
2020	38	67.1	2 (5.3%)	22 (57.9%)
2021	70	68.3	2 (2.9%)	16 (22.9%)
2022	64	64.3	12 (18.8%)	34 (53.1%)
2023	56	68.2	3 (5.4%)	20 (35.7%)

Abbreviations: N, number of cases; n, number; %, percentage.

## Data Availability

The data presented in this study are available on request from the corresponding author.
